# Geometric calibration of a stationary digital breast tomosynthesis system based on distributed carbon nanotube X-ray source arrays

**DOI:** 10.1371/journal.pone.0188367

**Published:** 2017-11-29

**Authors:** Changhui Jiang, Na Zhang, Juan Gao, Zhanli Hu

**Affiliations:** 1 Lauterbur Research Center for Biomedical Imaging, Shenzhen Institutes of Advanced Technology, Chinese Academy of Sciences, Shenzhen, China; 2 Shenzhen College of Advanced Technology, University of Chinese Academy of Sciences, Shenzhen, China; Sichuan University, CHINA

## Abstract

Stationary digital breast tomosynthesis (sDBT) with distributed X-ray sources based on carbon nanotube (CNT) field emission cathodes has been recently proposed as an approach that can prevent motion blur produced by traditional DBT systems. In this paper, we simulate a geometric calibration method based on a proposed multi-source CNT X-ray sDBT system. This method is a projection matrix-based approach with seven geometric parameters, all of which can be obtained from only one projection datum of the phantom. To our knowledge, this study reports the first application of this approach in a CNT-based multi-beam X-ray sDBT system. The simulation results showed that the extracted geometric parameters from the calculated projection matrix are extremely close to the input values and that the proposed method is effective and reliable for a square sDBT system. In addition, a traditional cone-beam computed tomography (CT) system was also simulated, and the uncalibrated and calibrated geometric parameters were used in image reconstruction based on the filtered back-projection (FBP) method. The results indicated that the images reconstructed with calibrated geometric parameters have fewer artifacts and are closer to the reference image. All the simulation tests showed that this geometric calibration method is optimized for sDBT systems but can also be applied to other application-specific CT imaging systems.

## Introduction

Traditional digital breast tomosynthesis (DBT) systems have been widely used, as such systems can overcome the overlapping phenomenon associated with mammography and can allow for differentiation between normal and pathological tissues on standard 2D projections in clinical settings[1–3]. However, the performance of traditional DBT is flawed; in particular, during a single scan, the X-ray tube moves along an arc and acquires few 2D projections within a limited angle[4]. This movement causes instability in the system gantry, resulting in the appearance of severe artifacts in the reconstructed computed tomography (CT) images. In recent years, stationary DBT (sDBT) systems have been proposed to solve this problem. Qian *et al*. reported a sDBT system that uses a carbon nanotube (CNT)-based multi-beam field emission X-ray (MBFEX) source[5]. Quan *et al*. presented a multi-beam system that uses linear arrays of X-ray sources arranged in a square geometry[6]. Square sDBT systems include two arrays of CNT X-ray sources and two panel detectors in a rectangular arrangement. During one scanning cycle, the object phantom remains stationary, and neither the linear array sources nor the panel detectors are rotated. Using this approach and device can solve the motion artifact problem associated with traditional DBT scanners that is caused by rotation of the mechanical gantry during scanning. The use of square sDBT systems can significantly improve imaging quality. Other potential advantages of sDBT systems relative to other DBT systems include reduced total imaging time and simplified system design[7]. However, for such systems, errors during manufacturing and assembly are difficult to avoid due to the use of multiple CNT sources. These errors affect the final quality of the reconstructed images and can result in severe artifacts in CT images. Thus, to obtain the perfect geometric parameters for an imaging system, accurate geometric calibration is necessary[8]. Accurate geometric parameters are also crucial for high-quality image reconstruction by CT systems[9].

After decades of research and development, various geometric calibration methods have been used for different X-ray imaging systems[10]. These methods can be classified as analytic calibration methods[11–18] and iterative optimization calibration methods[19–23] based on the algorithm used during the process of geometric calibration. In iterative optimization calibration methods, CT system parameters are estimated by calculating coordinate values and iteratively revising parameters from original estimates using various penalty terms. In analytic calibration methods, reconstruction parameters are obtained by directly calculating the elliptical geometries formed by combining all of the projection images obtained at different scanning angles. Analytic calibration methods are more commonly used in the field of CT reconstruction, as such methods have many advantages, including that they are easier to implement and require fewer calculations.

Geometric calibration methods can also be divided into phantom-based methods[12,18,24] and phantom-less methods[25–27] according to whether a customized or universal phantom is used in the process of geometric calibration. In phantom-based methods, to estimate geometric parameters, a calibration phantom consisting of certain numbers of markers must be used to acquire projections. A phantom-based calibration method can obtain geometric parameters based on the relationships between projected locations and the predefined positions of markers. The accuracy of such methods depends on the manufacturing precision of the calibration phantom. In phantom-less methods, no calibration phantom is utilized during the calibration process. Instead, geometric parameters are directly calculated from projection images, although these approaches involve expensive computational loads.

Each of the aforementioned methods has advantages and disadvantages. In this article, we report a simulation study of geometric calibration with a phantom-based method for a proposed multi-source CNT X-ray sDBT system. The method is a projection matrix-based approach in which only one projection datum of the phantom at an arbitrary incidence angle is required to obtain all geometric parameter information for the sDBT system with CNT X-ray sources and flat-panel detectors.

This paper is structured as follows. The methods and materials section provides a detailed description of a generic geometric calibration method based on projection matrices. In the results section, this method is applied to extract system geometric parameters of a SDBT system, and the geometric parameters were compared. To more intuitively test the accuracy of the geometric algorithms, a traditional cone-beam CT (CBCT) system with installation error was also simulated. During the calibration process, a self-fabricated phantom with 8 ball markers was utilized to extract the geometric parameters of the CBCT system, and a modified Shepp-Logan phantom was used to obtain the projection data. Finally, the projection data was reconstructed based on a universal filtered back projection (FBP) algorithm with and without the calibrated geometrical parameters to evaluate the efficacy of the geometric calibration algorithm. In the conclusion section, the major findings of the study are summarized.

## Methods and materials

**Fig 1:**
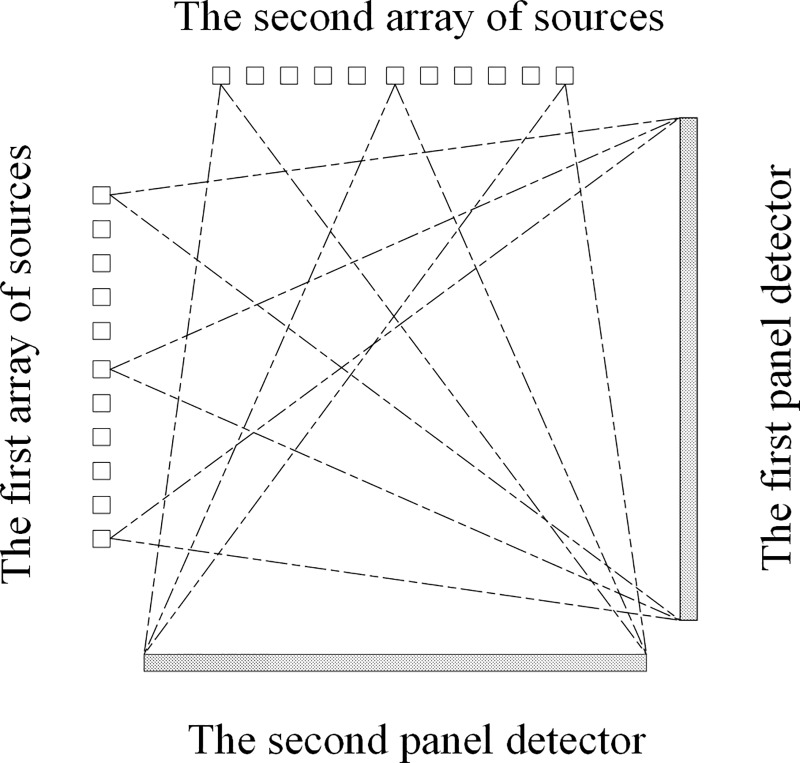
Schematic representation of the imaging geometry, with two linear arrays of CNT sources arranged opposite the detectors to form the rectangular sDBT system.

In the simulation, the geometry of the examined sDBT system is composed of two linear arrays of CNT X-ray sources and two panel detectors. These four components form a square, as illustrated in Fig 1.

**Fig 2:**
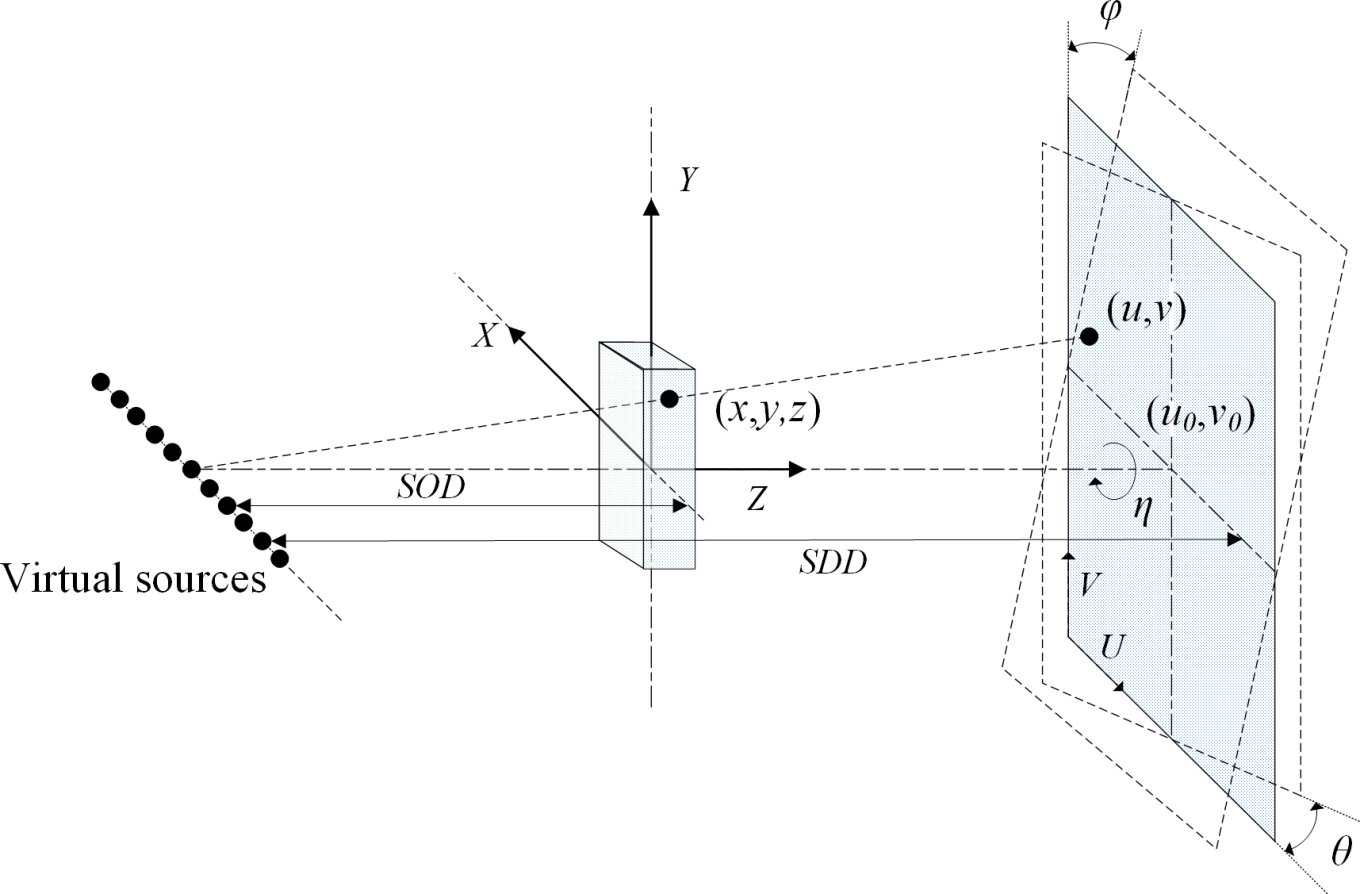
Schematic of the projection and coordinate system.

First, we specify seven important parameters of the sDBT system for image reconstruction (Fig 2): (*u*_*0*_, *v*_*0*_), the coordinates of the orthogonal projection of an X-ray focal spot on the detector plane; SOD, the distance from a source to the center of the square; SDD, the source-to-detector distance; *η*, the rotation angle of the detector plane along its normal vector; *φ*, the rotation angle of the detector plane along the *u* = *u*_*0*_ axis; *θ*, the rotation angle of the detector plane along the *v* = *v*_*0*_ axis; (Xs, Ys, Zs), the coordinates of the sources; and (*u*, *v*), the coordinates of the sources’ projections on the detector.

These seven parameters are determined using the formulae described below. For additional details, please see reference [28]. In this square sDBT system (see Fig 1), the calibration process is identical for all CNT X-ray sources; therefore, we can consider one CNT source as an example to illustrate the geometric calibration principle.

A projection matrix is a 3 × 4 matrix that relates the mapping of a point (*x*, *y*, *z*) in object coordinates to its projection (*u*, *v*) on a two-dimensional detector defined using homogeneous coordinates:

$${\left[Ax,Ay,Az,A\right]}^{T}={\left[au,av,a\right]}^{T}$$ (1)

where α is an arbitrary scaling factor. The projection matrix *A* can be factorized as:

A=K[S|t] (2)

where *K* is a 3 × 3 upper triangular matrix, *S* is a 3 × 3 rotation matrix, and *t* is a 3 × 1 translation vector.

$$K=\left[\begin{array}{ccc} \frac{SDD}{\lambda } & 0 & u_{0}\\ 0 & \frac{SDD}{\lambda } & v_{0}\\ 0 & 0 & 1 \end{array}\right]$$ (3)

where *u*_0_ and *v*_0_ are the coordinates of the intersection point associated with the central ray and the detector, and *λ* is the detector pixel size. The parameter *S* can be further represented using three Euler angles or a unitary quaternion:

$$S=\left[\begin{array}{ccc} \cos\eta \cos\varphi & \sin\theta \cos\eta \sin\varphi -\cos\theta \sin\eta & \sin\varphi \cos\theta \cos\eta +\sin\eta \sin\theta \\ \sin\eta \cos\varphi & \sin\theta \sin\eta \sin\varphi +\cos\theta \sin\eta & \sin\eta \cos\theta \cos\varphi -\cos\eta \sin\theta \\ -\sin\varphi & \cos\varphi \sin\theta & \cos\varphi \cos\theta \end{array}\right]$$ (4)

The three Euler angles *η*, *φ* and *θ* represent the orientation of the detector plane in the object frame. In formula (2), parameter *t* is:

$$t=\left[\begin{array}{l} t_{1}\\ t_{2}\\ t_{3} \end{array}\right]$$ (5)

Thus, *u*_0_ and *v*_0_ can be expressed explicitly. From formula (3):

$$u_{0}=K_{13},\;v_{0}=K_{23}$$ (6)

The parameter source-to-object distance (*SOD*) is:

$$SOD=K_{11}\lambda$$ (7)

The rotation angles of the detector are:

$$\begin{array}{l} \theta =\mathrm{Arc}\;\tan2(S_{32},S_{33})\\ \varphi =\sin^{-1}(-S_{31})\\ \eta =\mathrm{Arc}\;\tan2(S_{12},S_{11}) \end{array}$$ (8)

The source position is:

$$o={\left[o_{x},o_{y},o_{z}\right]}^{T}=-S^{T}t$$ (9)

where *t* is:

$$\begin{array}{l} t_{1}=A_{34}\\ t_{2}=(A_{24}-K_{23}A_{34})/K_{22}\\ t_{3}=(A_{14}-t_{13}A_{34}-K_{2}A_{12})/K_{11} \end{array}$$ (10)

Thus, the SOD is:

$$SOD=\sqrt{{\left(o_{y}\right)}^{2}+{\left(o_{x}\right)}^{2}+{\left(o_{z}\right)}^{2}}$$ (11)

**Fig 3:**
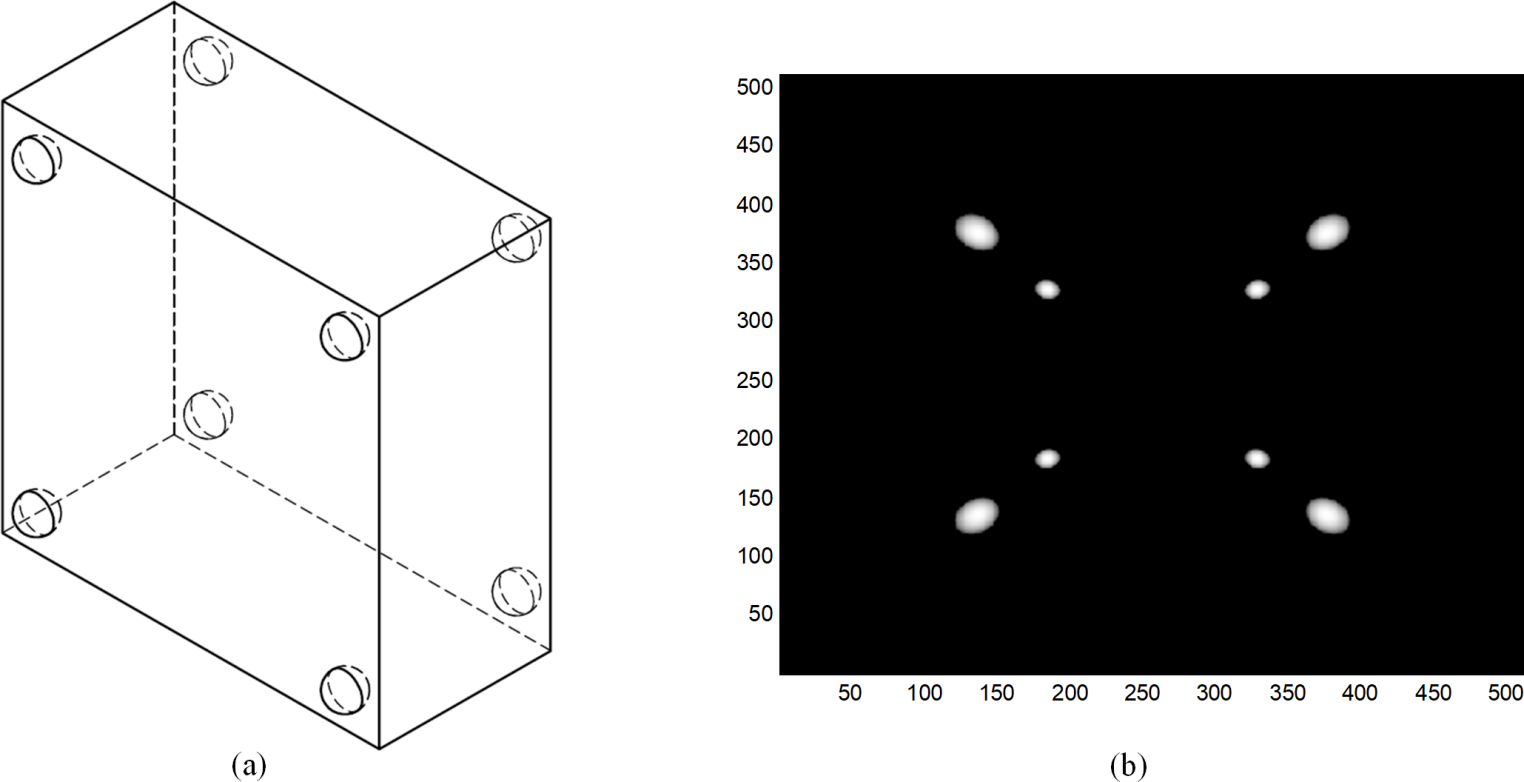
(a) Schematic of the calibration phantom. (b) Projection of the calibration phantom for individual emission by one of the CNT X-ray sources located in the middle.

To extract geometric parameters of the sDBT system, we designed a calibration phantom that contains 8 ball markers, each of which has a diameter of 10 units. In a realistic system, there are many possible arrangements of these markers in the phantom. To simplify the implementation of our calibration method, we designed the calibration phantom as shown in Fig 3(A). The ball markers are arranged in two parallel planes with four balls in each plane, and the balls’ coordinates and geometric parameters are known. To accurately calculate the seven geometric parameters mentioned above, it is extremely important to determine the exact center coordinates of these marker balls on the projection images. We use MATLAB programs to obtain the centers of the ball markers in the projection images via an approach based on a least squares and genetic algorithm. We then calculate the projection matrix of the square sDBT system for each CNT X-ray source-panel detector pair. Finally, the aforementioned calibration method based on a computer simulation is presented with a calibration phantom using Visual C++.

Specifically, the proposed geometric calibration method is implemented via the following steps. First, the phantom mentioned above is imported into the simulation model to imitate the acquisition of projection data. In the square sCBT system, which has 22 CNT X-ray sources distributed across two linear arrays, each CNT X-ray source is individually controlled, and sources are switched on one by one. That is, at each time, only one source emits X-rays, and the detector acquires one projection image, as illustrated in Fig 3(B). In these projection images, the projections of the ball markers are elliptical. The center coordinates of these ellipses in the detector plane are then extracted. Finally, the projection matrix is calculated based on the mapping relationship between the known 3D coordinates of point markers and the 2D projection coordinates of these markers in the image plane. The seven aforementioned geometric parameters are then derived.

## Results

### Extracted geometrical parameters of the sDBT system and results analysis

For this simulation, we define all lengths in units of detector pixels. To reduce calculation requirements, each source array only contains 11 CNT X-ray sources that are equidistantly distributed across the linear array. The two arrays form two contiguous sides of a square in the transaxial plane. The length of the source array is 100, and two 512 (width) × 512 (height) flat-panel detectors form the other two sides of the square. The source-to-detector distance (SDD) is 200 units, and the SOD is 100 units. A coordinate system is defined as illustrated in Fig 2. During a scan, the object phantom remains stationary, and neither the two linear array sources nor the two panel detectors rotate.

**Fig 4:**
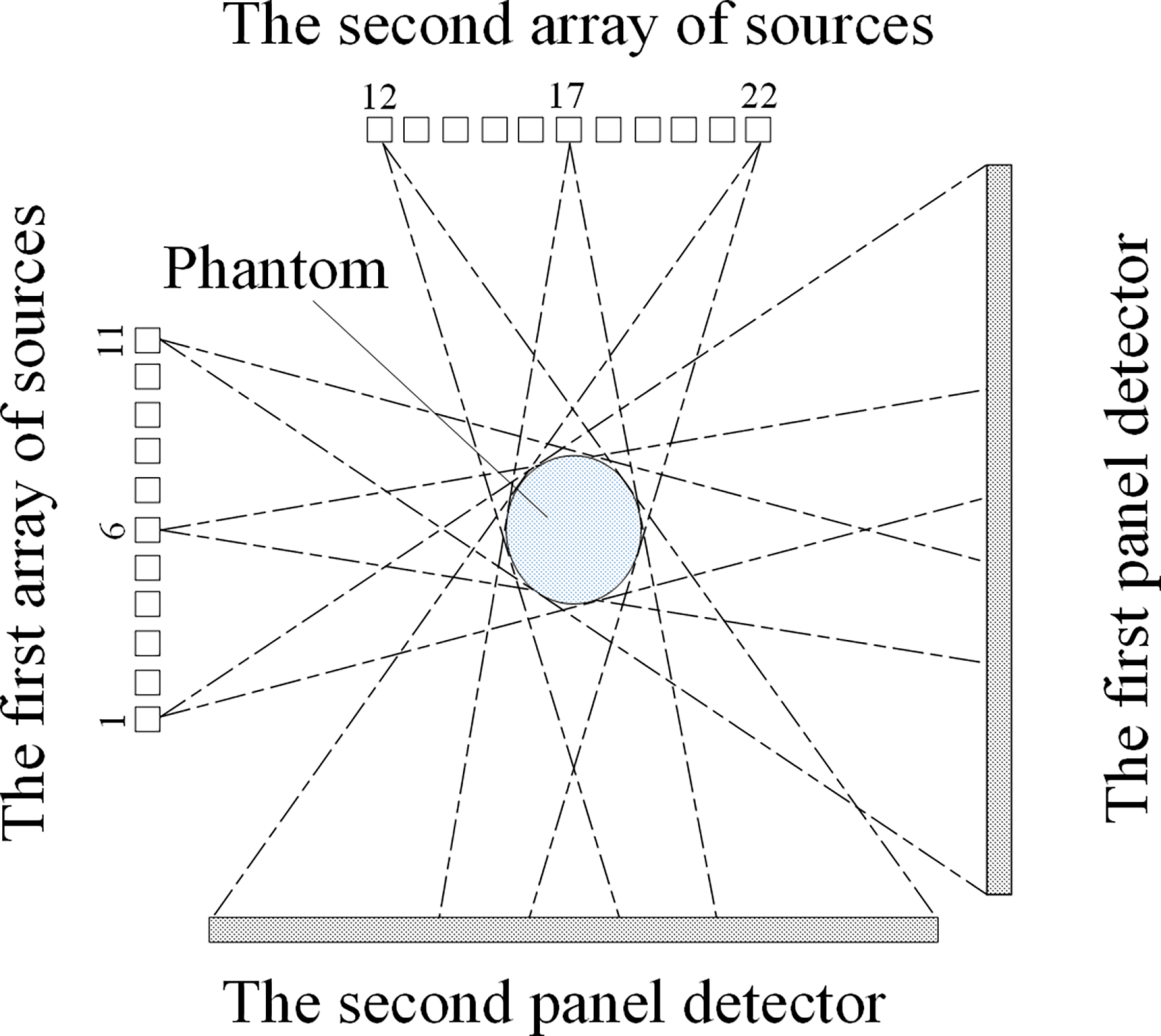
Schematic representation of the simulation projection process; the 22 CNT X-ray sources are numbered (1–22) and are sequentially triggered for emission.

**Fig 5:**
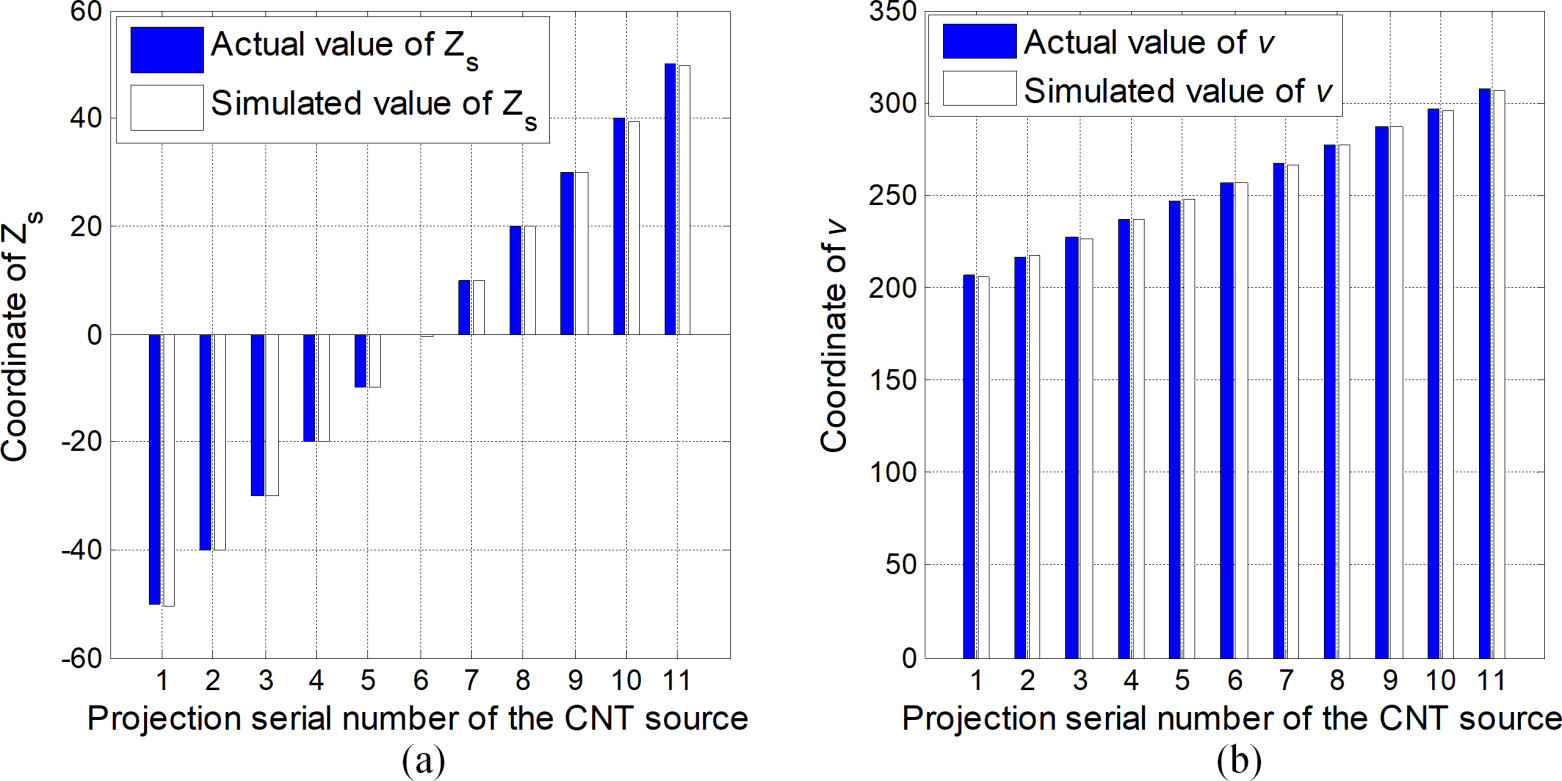
Comparisons of input values and extracted values of the coordinates of the CNT sources (Zs) (a) and the coordinates of the sources’ projections (*v*) (b).

To test the accuracy of the aforementioned geometrical calibration method, the phantom is simulated in the square sDBT system, as illustrated in Fig 4. We utilize the projection’s 2D coordinates on the image detector and the previously known 3D coordinates of the ball markers to compute the geometric parameters. To demonstrate the results of the geometric calibration, the geometric parameters that are input into the simulation are compared with the extracted parameters. Because the calibration mechanisms of the two arrays of sources are completely identical, we can focus on the geometric calibration of the first array of sources and the corresponding detector. We take the 6th X-ray source (Fig 4) as an example to demonstrate the calibration process. The input parameters and extracted parameters for each CNT source are shown in Fig 5 and Table 1.

**Table 1:** Comparisons of the input geometric parameters and the extracted simulation parameters.

		*φ* (deg)	*θ* (deg)	*η* (deg)	X_s_	Y_s_	SDD	SOD	*u*
**Input parameters**		0	0	90	0	-100	200	100	257
**Extracted parameters**	**1**	0.0472	0	90.0002	-0.463561	-99.2268	198.763	99.2268	256.258
	**2**	0.0397	0	90.0002	-0.466712	-99.8267	200.11	99.8563	256.25
	**3**	0.0562	0.1825	90.0002	-0.463561	-99.2268	198.763	99.2268	256.258
	**4**	0.0216	0	90.0002	-0.463561	-99.2268	198.763	99.2268	256.258
	**5**	0.0563	0	90.0002	-0.463561	-99.2268	198.763	99.2268	256.258
	**6**	0	0	90.0002	-0.463561	-99.2268	198.763	99.2268	256.258
	**7**	-0.011	0	90.0002	-0.463561	-99.2268	198.763	99.2268	256.258
	**8**	0	0	90.0002	-0.463561	-99.2268	198.763	99.2268	256.258
	**9**	-0.1738	-0.0002	90.0002	-0.463561	-99.2268	198.763	99.2268	256.258
	**10**	0.1287	0	90.0002	-0.463561	-99.2268	198.763	99.2268	256.258
	**11**	-0.0098	0.1591	90.0002	-0.463561	-99.2268	198.763	99.2268	256.258

Fig 5 and Table 1 indicate that the extracted geometric parameters are extremely close to the input values, and most of the errors for the obtained values are less than 1 detector pixel unit, except the errors of the SDD. Moreover, the maximum error for the SDD is 1.2 pixel units, which is much smaller than the errors associated with actual mechanical installation. In this computer simulation experiment, the results obtained using the calibration method are extremely close to the corresponding actual values.

**Fig 6:**
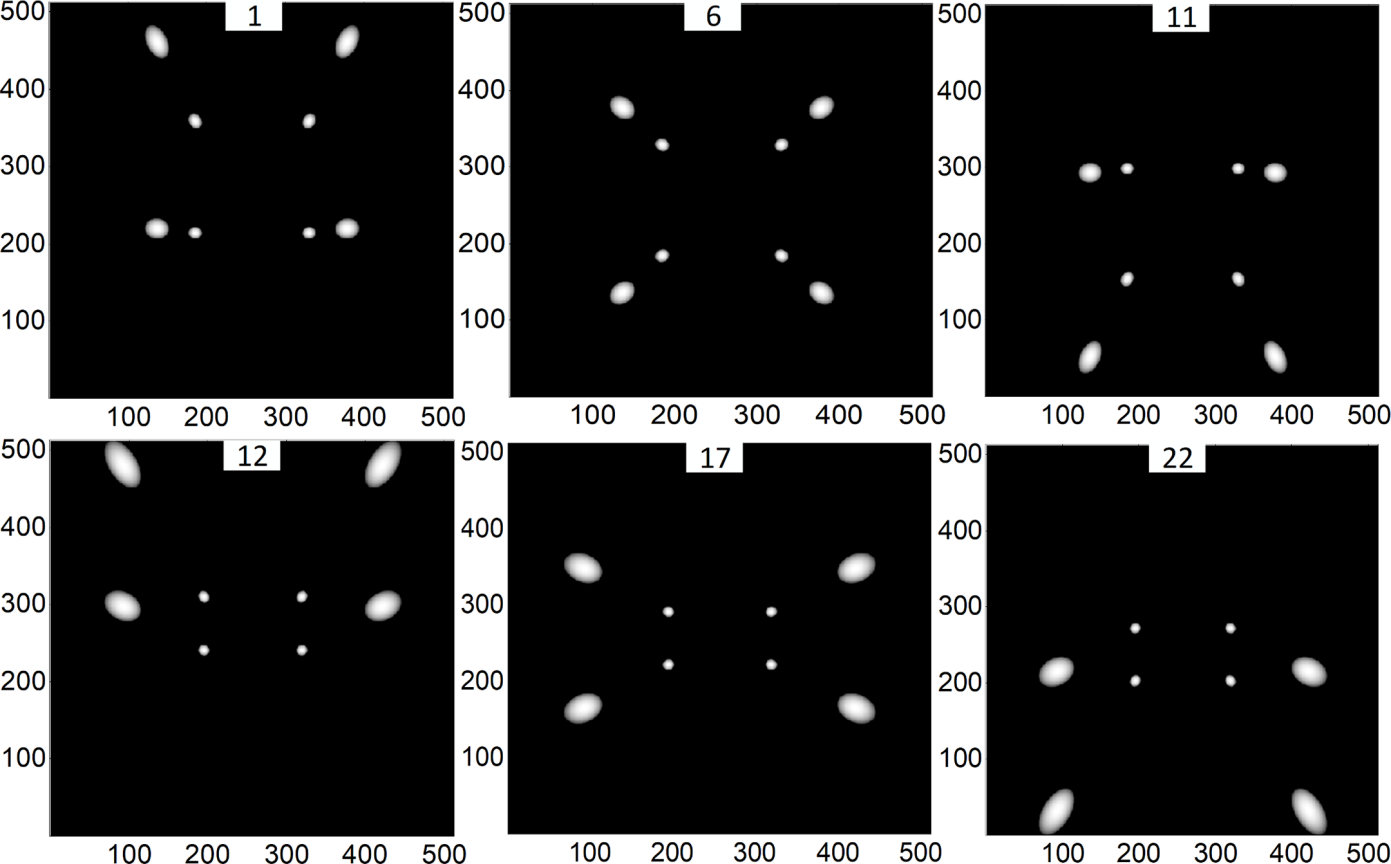
Projections of the calibration phantom for all CNT X-ray sources, taking the projections of the 1st, 6th, 11th, 12th, 17th and 22nd source (see Fig 4) as examples.

By repeating the above steps, we calculate the geometric parameters of the remaining CNT X-ray sources. The projections of the calibration phantom for these 22 CNT X-ray sources are shown in Fig 6. Finally, we can obtain all the geometric parameters of the square sDBT system.

### Simulation tests of traditional cone-beam CT

The input geometric parameters and the extracted parameters are compared above. The experiments demonstrate that the extracted geometric parameters are quite close to the input values. To more intuitively test the accuracy of the geometric calibration algorithm, traditional CBCT with a flat-detector was simulated. The source and detector rotated synchronously while a stationary object was scanned. The SOD was set as 570 mm and the SDD as 1040 mm. The size of the flat-detector was 512×512. To simulate the installation error of the detector, the center of the detector was installed offset by 0, 1, 2, or 5 pixels.

The calibration process of the CBCT was as follows. First, a self-fabricated calibration phantom with 8 ball markers were utilized to extract the geometric parameters of the CBCT system, and four sets of geometric parameters were obtained. Then, a modified Shepp-Logan phantom is used to obtain the projection data during each scan, and four sets of projection data were obtained according to these four sets of geometric parameters. Finally, the projection data were reconstructed based on a universal filtered back projection (FBP) algorithm with and without the calibrated geometrical parameters to evaluate the efficacy of the geometric calibration algorithm.

As shown in Table 2 and Table 3, all the input geometric parameters and extracted parameters of the CBCT system are highly similar, except SDD, SOD and Y_s_. In the simulated CBCT system, these three parameters described the distances between the X-ray source, the object and the detector. Although these three parameters seemed to have larger errors than other parameters, these errors empirically have limited influence on reconstructed images.

**Table 2:** Comparisons of the set geometric parameters and the extracted simulation parameters.

	*φ*	*θ*	*η*	X_s_	Y_s_	Z_s_	SDD	SOD
	(deg)	(deg)	(deg)	(mm)	(mm)	(mm)	(mm)	(mm)
**set parameters**	0	0	90	0	-570	0	1040	570
**0-pixel offset**	0	0	90.0002	-0.00005	-565.385	-0.00005	1030.77	565.385
**1-pixel offset**	0	0	90.0002	0.00032	-565.385	-0.00005	1030.77	565.385
**2-pixel offset**	0	0	90.0002	-0.00028	-565.385	-0.00005	1030.77	565.385
**5-pixel offset**	0	0	90.0002	-0.00013	-565.385	-0.00005	1030.77	565.385

**Table 3:** Comparisons of the set geometric parameters and the extracted simulation parameters of the coordinates of the sources’ projections on the detector. (*u*_set_, *v*_set_) and (*u*_ext_, *v*_ext_) represent the set and extracted coordinates, respectively, of the sources’ projections on the detector.

	*u*_*set*_	*u*_*ext*_	*v*_*set*_	*v*_*ext*_
**0-pixel offset**	257	257	257	257
**1-pixel offset**	258	258	257	257
**2-pixel offset**	259	259	257	257
**5-pixel offset**	262	262	257	257

After the geometric parameters of the CBCT system were obtained, a modified 3D Shepp-Logan phantom was scanned, and 360 equally-spaced projections were obtained in one scanning circle. These projections were then used for image reconstruction based on the FBP method with the calibrated and uncalibrated geometric parameters.

**Fig 7:**
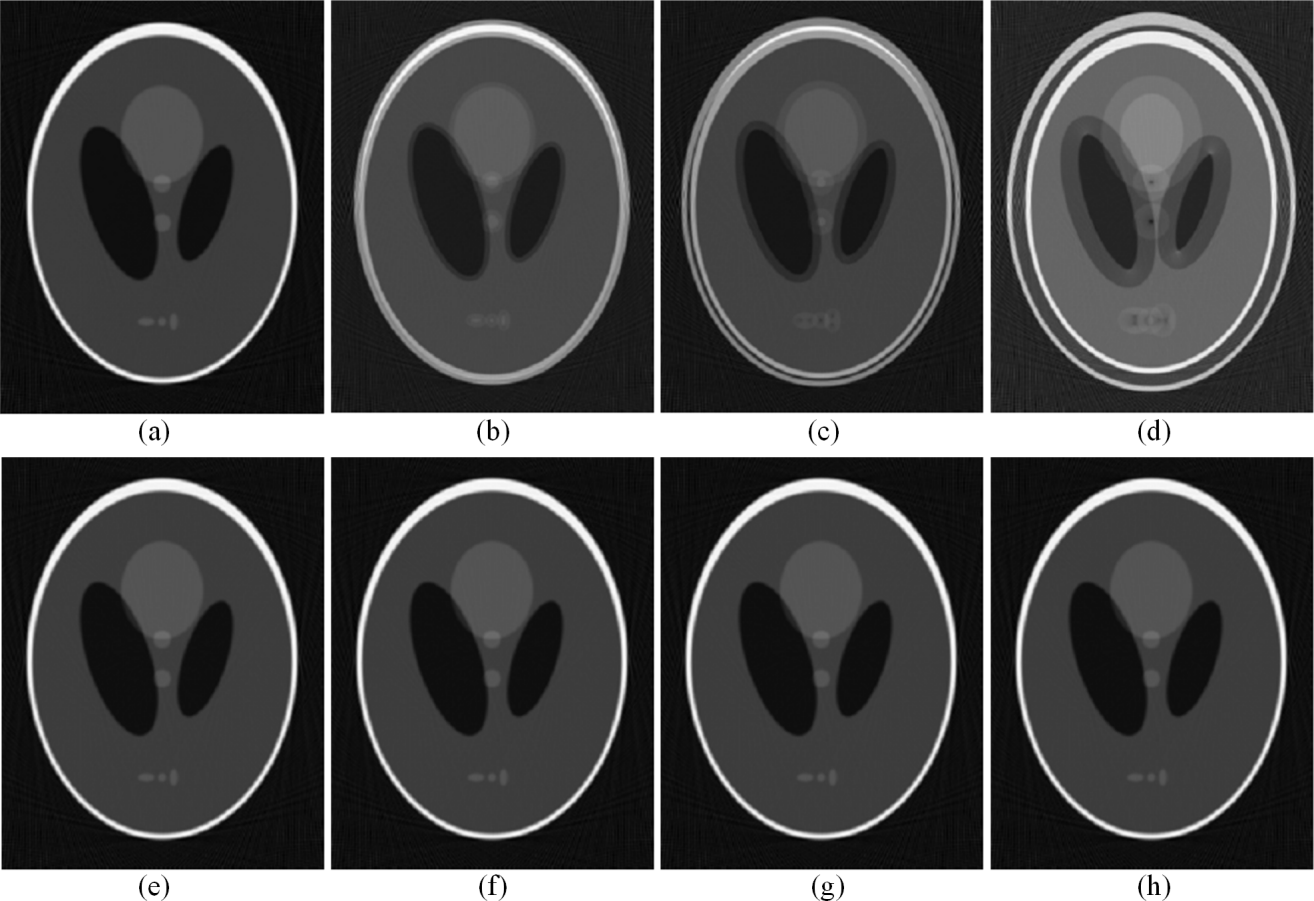
Reconstructed images using geometrical parameters with and without calibration. (a) A reconstructed image with input geometrical parameters without offset. [(b)-(d)] Reconstructed images with different offsets but without calibration. (b) offset = 1 pixel; (c) offset = 2 pixels; (d) offset = 5 pixels. (e) A reconstructed image with extracted geometrical parameters without offset. (f) The reconstructed image in (b) after calibration. (g) The reconstructed image in (c) after calibration. (h) The reconstructed image in (d) after calibration.

Four different CBCT systems with different mechanical installation errors were simulated. The FBP-reconstruction results of the modified Shepp-Logan phantom are shown in Fig 7. Images (a)~(d) of Fig 7 were reconstructed with the input geometrical parameters without calibration, and obvious artifacts were observed when the installation error was introduced into the imaging system. The reconstructed image for the 1-pixel offset is very blurred, the reconstructed image for the 2-pixel offset contains severe artifacts, and it is nearly impossible to distinguish any structural information of the reconstructed image for the 5-pixel offset. Images (e)~(h) were reconstructed with calibrated geometrical parameters, and the artifacts in these images caused by installation errors were effectively suppressed.

**Fig 8:**
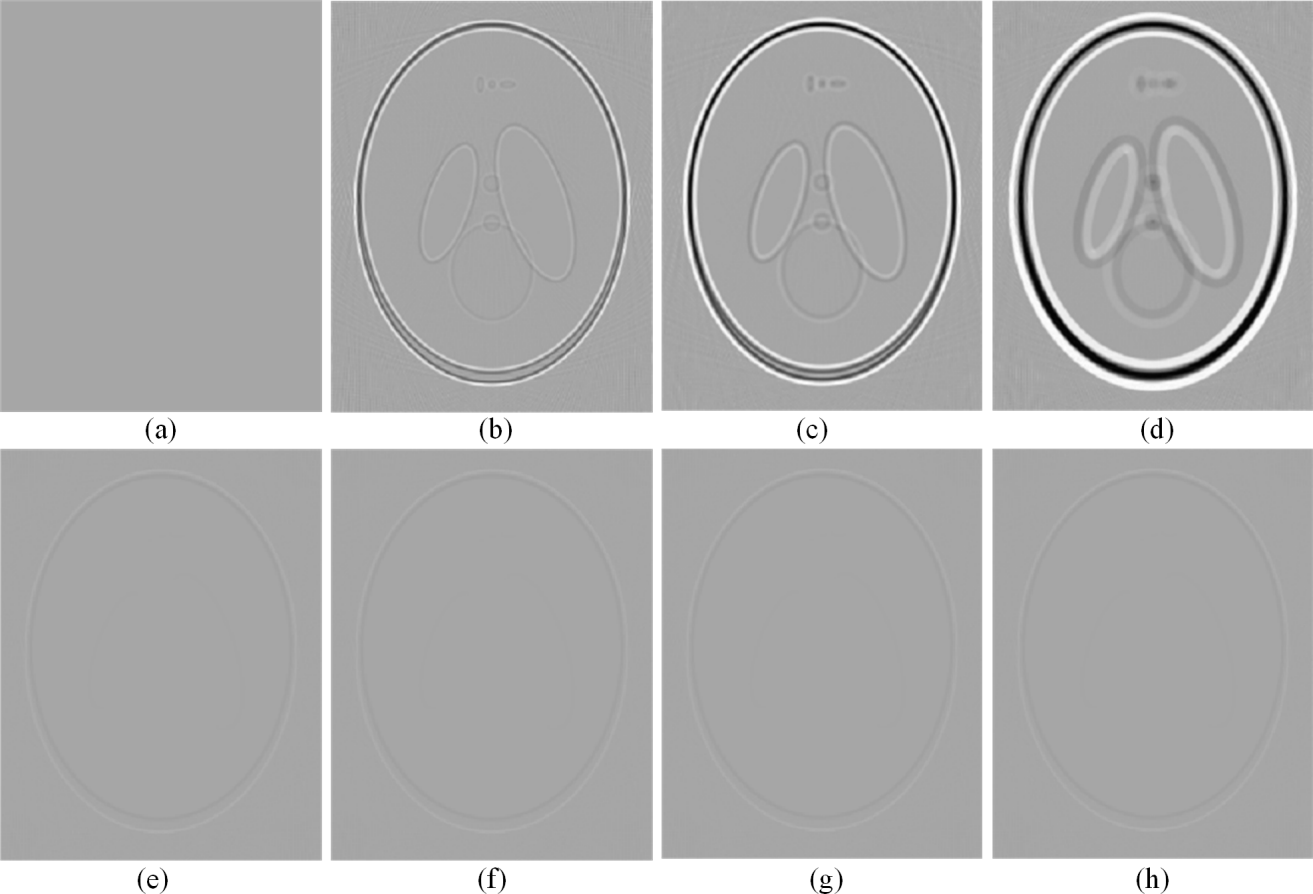
The residual images of the reconstructed results shown in Fig 7. The images are displayed in the window [-0.13 0.07].

**Fig 9:**
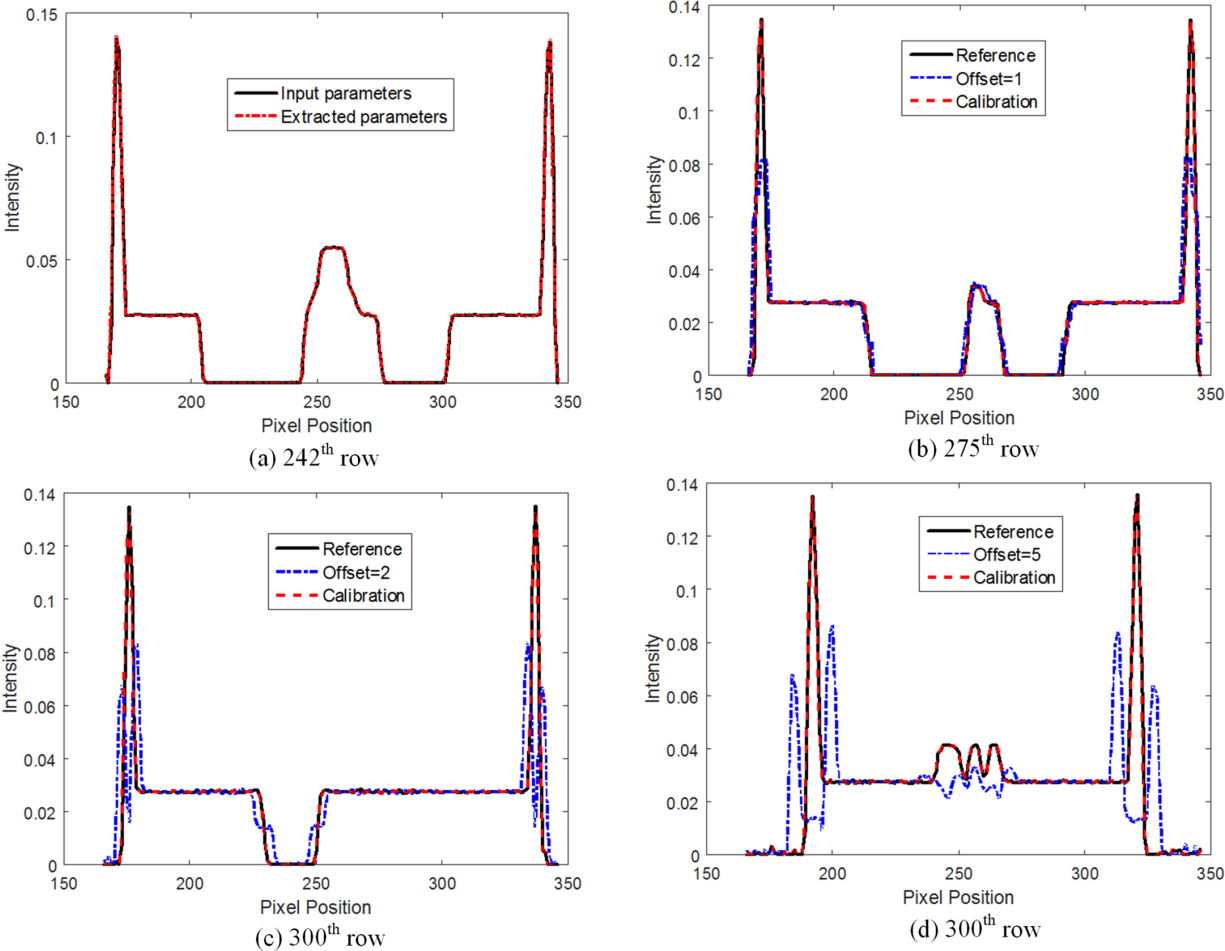
Profiles of different results shown in Fig 7. (a) Profile of the reconstructed images with input or extracted geometrical parameters without offset in the 242^th^ row. (b) Profile of the reconstructed images with or without geometrical calibration with 1-pixel offset in the 275^th^ row. (c) Profile of the reconstructed images with or without geometrical calibration with 2-pixel offset in the 300^th^ row. (d) Profile of the reconstructed images with or without geometrical calibration with 5-pixel offset in the 335^th^ row.

The image shown in Fig 7 (A) is a reconstructed image with input geometrical parameters and without offset, which was considered as the reference image. To quantitatively evaluate the reliability of the proposed algorithm, the profile images of the reconstructed images and the residual images with the reference image are shown in Fig 8 and Fig 9, respectively. The results show that the proposed method can help achieve a superior image quality after reconstruction without calibration in terms of preserving the structure and suppressing undesired artifacts, which indicates its useful potential for CT imaging.

## Conclusions

We developed a geometric calibration method based on a projection matrix approach for a square sDBT system and verified its performance. The errors of the input geometric parameters and the extracted parameters are compared based on the results of the geometric parameters extracted from the sDBT system. The simulations demonstrated that the extracted geometric parameters are quite close to the input values. Furthermore, to more intuitively test the accuracy of the geometric algorithms, traditional scanning in a circular manner using the CBCT system with a flat-detector was simulated. The reconstructed images with and without calibrated geometrical parameters were compared. The results indicated that the proposed algorithm can be used to extract the geometric parameters with sufficient accuracy for image reconstruction and can significantly reduce the artifacts caused by installation errors. In conclusion, the proposed calibration method not only can be used to extract the seven geometric parameters of the square sDBT system but can also be used in other traditional X-ray imaging systems, and these parameters were consistent with the corresponding actual input values with high numerical precision.

## Supporting information

S1 File The code of this manuscript. The datasets generated or analyzed during the current study are available at https://figshare.com/s/96d8a1ef9c537f74411c.
(ZIP)
